# Retraction Note: Human amniotic membrane grafts for retinal breaks in diabetic tractional retinal detachment and combined tractional and rhegmatogenous retinal detachment

**DOI:** 10.1038/s41598-022-08884-9

**Published:** 2022-03-23

**Authors:** Yen-Chih Chen, San-Ni Chen

**Affiliations:** 1 Department of Ophthalmology, Changhua Christian Hospital, No. 135, Nanxiao Street, Changhua City, Taiwan; 2 Department of Ophthalmology, Yunlin Christian Hospital, Xiluo, Taiwan; 3 Department of Optometry, Central Taiwan University of Science and Technology, Taichung City, Taiwan; 4 School of Medicine, Chung-Shan Medical University, Taichung, Taiwan; 5 School of Medicine, Kaohsiung Medical University, Kaohsiung, Taiwan; 6 Department of Optometry, Da-Yeh University, Changhua, Taiwan

Retraction of: *Scientific Reports* https://doi.org/10.1038/s41598-021-86804-z, published online 13 April 2021

The Editors have retracted this Article.

Following the publication of this Article it has been brought to the Editors' attention by the Changhua Christian Hospital that the authors did not have relevant ethical approvals prior to commencing the study.

Both authors disagree with this retraction.

